# The power of combined modalities in interactive robot learning

**DOI:** 10.3389/frobt.2025.1598968

**Published:** 2025-07-17

**Authors:** Helen Beierling, Robin Beierling, Anna-Lisa Vollmer

**Affiliations:** Interactive Robotics in Medicine and Care, Medical School OWL, Bielefeld University, Bielefeld, Germany

**Keywords:** human-robot interaction, human-in-the-loop learning, reinforcement learning, interactive robot learning, multi-modal feedback, learning from demonstration, preference-based learning, scaffolding in robot learning

## Abstract

With the continuous advancement of Artificial intelligence (AI), robots as embodied intelligent systems are increasingly becoming more present in daily life like households or in elderly care. As a result, lay users are required to interact with these systems more frequently and teach them to meet individual needs. Human-in-the-loop reinforcement learning (HIL-RL) offers an effective way to realize this teaching. Studies show that various feedback modalities, such as preference, guidance, or demonstration can significantly enhance learning success, though their suitability varies among users expertise in robotics. Research also indicates that users apply different scaffolding strategies when teaching a robot, such as motivating it to explore actions that promise success. Thus, providing a collection of different feedback modalities allows users to choose the method that best suits their teaching strategy, and allows the system to individually support the user based on their interaction behavior. However, most state-of-the-art approaches provide users with only one feedback modality at a time. Investigating combined feedback modalities in interactive robot learning remains an open challenge. To address this, we conducted a study that combined common feedback modalities. Our research questions focused on whether these combinations improve learning outcomes, reveal user preferences, show differences in perceived effectiveness, and identify which modalities influence learning the most. The results show that combining the feedback modalities improves learning, with users perceiving the effectiveness of the modalities vary ways, and certain modalities directly impacting learning success. The study demonstrates that combining feedback modalities can support learning even in a simplified setting and suggests the potential for broader applicability, especially in robot learning scenarios with a focus on user interaction. Thus, this paper aims to motivate the use of combined feedback modalities in interactive imitation learning.

## 1 Introduction

The growing sophistication of artificial intelligence (AI) has expanded the market for autonomous systems, such as autonomous cars and vacuum robots. There is a growing demand for intelligent agents that can simplify everyday tasks, particularly in home environments or care. These areas of application are characterized by diverse individual preferences and tasks. To address this, users, especially lay users, need to be empowered to teach robots new actions according to their preferences. Interactive imitation learning, including human-in-the-loop reinforcement learning (HIL-RL), with active user feedback, is a common method to realize this teaching Celemin et al. (2022). Human feedback in HIL-RL scenarios is valuable, as reward functions normally designed to provide customized learning feedback for specific situations may underperform in different contexts Kaufmann et al. (2023). Furthermore, it can be challenging to rationally define such functions via reward engineering, especially for tasks based on personal preferences (Kaufmann et al., 2023; Knox et al., 2023). There is a variety of literature spanning from older approaches (Isbell et al., 2001; Knox and Stone, 2008) to more recent ones (Hindemith et al., 2022; Ding et al., 2023) regarding the use of human feedback that were able to show positive results with various human feedback modalities. Thus, incorporating human feedback in HIL-RL is current and relevant, particularly in the field of robotics in everyday life scenarios.

However, when robots are taught by users through direct feedback, users naturally want to scaffold the learning robot. Originating in developmental psychology (Wood et al., 1976; Stone 1998a), scaffolding involves adjustments to the learning environment and providing guidance, constantly adapting to the progression of learning (Vollmer et al., 2016; Saunders et al., 2006; Jumaat and Tasir, 2014; Stone, 1998b; Breazeal, 1998). This scaffolding behavior also showed the potential to be valuable in robot teaching as well (Angeli and Valanides, 2020; Breazeal, 1998; Breazeal et al., 2006). Nevertheless, users tend to give, for example, overly positive ratings to push learning behavior in that direction due to their scaffolding intent, unknowingly distorting feedback and hindering learning (Thomaz and Breazeal, 2007; Thomaz and Cakmak, 2009; Celemin et al., 2022). This can be mitigated, for example, by providing guidance as a separate form of feedback modality. Thus, the literature advocates modalities that allow users to express this supplementary feedback to enhance feedback quality and guide actions effectively (Thomaz and Breazeal, 2006; Liu et al., 2023; Li et al., 2021; Bajcsy et al., 2018; Celemin and Kober, 2023; Ravichandar et al., 2020; Arzate Cruz and Igarashi, 2020; Celemin et al., 2022). This allows users, for instance, to mark actions as guidance, which are then functioning as a point of orientation for the ongoing learning process.

Although the literature presents various such beneficial modalities, they have largely only been tested in isolation or as a switching option between two modalities (Celemin et al., 2022; Casper et al., 2023), such as switching between demonstration and preference as a feedback modality Bıyık et al. (2022). However, the combination of different feedback modalities, the selection of multiple modalities at once, and their simultaneous use remain open challenges (Celemin et al., 2022; Arzate Cruz and Igarashi, 2020; Li et al., 2019). Therefore, we conducted a study to explore the interaction behavior of lay users in a real robot teaching scenario when a variety of feedback modalities is available to them at once. We were especially interested in the questions whether all modalities are utilized and perceived equally or whether there are usage and perception differences. This is particularly relevant because this knowledge would enable the identification of existing patterns from which the user’s mental model could be inferred, allowing the system to provide targeted explanations and support the user effectively. In addition, our goal was to determine whether their combined use offers a learning benefit to the system. This led us to the following four hypotheses for our study:

H1: Enhanced Learning through Multiple Modalities The learning success in a human-in-the-loop reinforcement learning framework can be enhanced by incorporating multiple modalities of direct user feedback.
H2: User Preferences and Modality Utilization Users do not utilize all modalities equally, but tend to show a preference for certain ones.
H3: Different Perception of Modalities Users subjectively perceive some to be more beneficial compared to others in contributing to learning success.
H4: Enhanced Learning through Individual Modalities The use of individual modalities is positively correlated with the success of the learning algorithm when they are employed.

## 2 Related work

In this section, we aim to provide an overview of approaches in Human-in-the-Loop robot training that are based solely on user evaluations. From this overview, we will derive a baseline modality. This baseline modality can then be extended with supplementary modalities, allowing us to compare the added value of combined modalities against the provision of the use of individual baseline modalities or simply switching between them. Additionally, we will present the rationale behind the selection of the supplementary modalities used.

There is a rich body of literature on the use of human feedback in reinforcement learning, spanning from early approaches (Isbell et al., 2001; Knox and Stone, 2008) to more recent advances (Hindemith et al., 2022; Ding et al., 2023). These studies demonstrate the effectiveness of human feedback across various modalities and application domains. As a result, incorporating human feedback into reinforcement learning remains an active and relevant research direction, particularly in the field of robotics.

For our baseline modality, we focus on the most widley used feedback modalities. Kaufmann et al. (2023) provide a comprehensive survey of reinforcement learning approaches that integrate direct user feedback, categorizing them into preference-based and reward-based learning methods. In both cases, users offer numerical or binary evaluations of an agent’s actions, constituting a form of evaluative feedback. Additionally, alternative modalities such as emotions (Yu and Tapus, 2019; Su et al., 2023), gestures (Su et al., 2023), or speech (Chen et al., 2022) can be utilized individually or in combination. However, this study specifically focuses on direct evaluative feedback methods.

Celemin et al. (2022) further distinguish imitation learning feedback modalities in robotics by classifying them into evaluative feedback modalities (assessing how well the robot performs) and transition-based feedback modalities (advising how the robot should act). Both types can be given in either a direct or indirect manner. Their findings indicate that relative evaluative feedback modalities are particularly well suited for lay users, as it aligns with expressing preferences over a robot’s actions (see Figure 3.5 in Celemin et al. (2022)).

This notion was also shown by Hindemith et al. (2022), who compared preference-based and scalar feedback modalities within a reinforcement learning framework in a human-robot interaction setup. Their study, which used user feedback as a direct reward in a cup-and-ball task performed by a Pepper robot, revealed that users found it easier to compare two trajectories rather than to provide scalar ratings, which required maintaining consistency with prior evaluations. Based on these insights, we adopt a preference-based feedback modality as the baseline modality for our study.

In addition to this, we identified six supplementary modalities from the literature that extend and complement this baseline:

### 2.1 Guidance

An earlier study by Thomaz and Breazeal (2006) highlights the importance of providing users with the option to provide information not only for present actions, but also for future actions, providing guidance to the robot. This method not only prevents users from misusing feedback modalities for guidance but also improves learning outcomes (Thomaz and Breazeal, 2007; Thomaz and Cakmak, 2009; Bignold et al., 2023; Celemin et al., 2022). Regarding the classification of Celemin et al. (2022) we refer to *guidance* as a relative evaluative feedback modality. Allow users to mark favored actions as guiding for future learning.

### 2.2 Correction

Correction is the widely used opposite of guidance (Liu et al., 2023; Chernova and Veloso, 2009; Meriçli et al., 2011; Casper et al., 2023; Celemin et al., 2022). The literature suggests that using corrections for human-in-the-loop robot learning also benefits learning (Liu et al., 2023; Li et al., 2021; Bajcsy et al., 2018; Celemin and Kober, 2023). Here we relate to corrections classified as relative corrections in state-action-space, not absolute corrections, referring to Celemin et al. (2022), meaning that users are able to mark actions as to be avoided instead of giving a direct example of the action.

### 2.3 Demonstration

An alternative to relative corrections is the option of demonste an action to the robot to assist or lead the learning process, which would be a direct correction (Celemin et al., 2022). This method is widely employed in robotics as in learning from demonstration approaches. One possible popular approach is kinesthetic teaching, in which the user directly manipulates the robot to demonstrate the action (Ravichandar et al., 2020; Meriçli et al., 2010; Casper et al., 2023; Celemin et al., 2022; Celemin and Kober, 2023).

### 2.4 Exploration

Exploration in learning algorithms refers to the process in which the algorithm tries different actions to discover which yield the best results (i.e., the highest reward). While the exploration versus exploitation dilemma is a long-standing challenge in machine learning, it becomes particularly pronounced in scenarios where the algorithm receives sparse feedback, such as human feedback. This dilemma involves deciding whether to try new approaches to discover better options or to stick with known strategies that yield the best results. This dilemma can be addressed by allowing users to directly control the exploration process by lowering or increasing the exploration rate (Arakawa et al., 2018; Arzate Cruz and Igarashi, 2020; Raffin et al., 2022; Torne et al., 2023; Thomaz and Cakmak, 2009).

### 2.5 Speed

A modality, closely aligned with the action advice, involves providing guidance on specific attributes of an action (Arzate Cruz and Igarashi, 2020; Argall et al., 2011; Celemin et al., 2022). Unlike providing absolute feedback in the state-action space based on an entire demonstration, this approach focuses on pinpointing individual attributes of an action perceived as suboptimal by the user. We selected execution speed as a relevant natural attribute for user advice.

### 2.6 Fallback

Fallback represents the option to revert to a previous state of action and resume learning from there. This modality is grounded in the concept of *Correction* or negative *Guidance*, as outlined by Thomaz and Breazeal (2006). In instances where the agent explores the action space and converges to undesired behavior, a emphFallback modality allows to retrace the explored path and to restart from the best action achieved thus far. This differs from *Correction*, where learning is only influenced according to the marked action, while the general direction of learning is maintained based on past positive and negative actions (Ecoffet et al., 2021).

Although there is considerable research on individual supplementary modalities, their combination remains an open question. A survey focusing on interactive robotics and human-robot interactions, examining trend-setting human feedback modalities, highlights the open challenge of combining feedback modalities and their application to reinforcement learning algorithms (Arzate Cruz and Igarashi, 2020). Similarly, the potential of combining feedback modalities in interactive reinforcement has been emphasized (Li et al., 2019; Celemin et al., 2022).

There are already studies exploring the use of multiple modalities in robot learning. However, these approaches typically rely on a human-determined switch between different modalities rather than leveraging supplementary modalities simultaneously.

Bıyık et al. (2022) combine demonstrations with preference-based feedback modality, showing that integrating both sources leads to faster and more accurate reward learning. Their method, *DemPref*, selects between demonstrations and preference queries, balancing efficiency and informativeness.

Another perspective comes from Jeon et al. (2020) and Jeon et al. (2020), who propose a unifying formalism to interpret various types of human feedback. They introduce the concept of Reward-Rational Implicit Choice (RRIC), where human behavior, whether demonstrations, corrections, comparisons, or even actions like turning off a robot, is viewed as a rational selection from an implicit set of choices. This formalism provides a general framework that encompasses a wide range of feedback modalities, helping to structure and unify reward inference across different modalities. While Jeon et al. (2020) model different feedback modalities as separate sources of reward information that are used sequentially or context-dependently, our work actively enables the simultaneous use of multiple modalities, allowing users to intuitively combine different feedback modalities in real time to influence the robot’s learning process. Furthermore, we validate our approach through an empirical user study, demonstrating the impact of multimodal feedback modalities on learning success and user perception and utilization.

By doing so, we address the current research gap on simultaneously offered combined modalities, positioning our work within state-of-the-art interactive robot learning while building upon and extending the approaches of the field.

## 3 Methods

### 3.1 Implementation

The modalities were implemented on a Kinova Gen2/Jaco (Jaco2) robotic arm Inc. K (2023) using the robotic operating system (ROS) Noetic Robotics (2020) on Ubuntu 20.04.

#### 3.1.1 Probabilistic movement primitives (ProMP)

To represent movements, we used an implementation of probabilistic movement primitives (ProMP) (Paraschos et al., 2013; Alexander Fabisch, 2020). ProMP depict movements based on parameterized distributions, making them highly adaptable as they cover an entire distribution of trajectories rather than singular trajectories. Moreover, they are notable for their compact design, which requires adjustments solely to their parameters to accommodate changes in all trajectories. The following parameter values are used for the ProMP implementation:

• The number of degrees of freedom $\left(n_{\text{dims}}=6\right)$ and the number of time steps per trajectory $\left(n_{\text{steps}}=20\right)$ define the motion structure.
•The weight dimension of the ProMP $\left(n_{\text{weights per dim}}=16\right)$ determines the number of parameters to optimize.
•The exploration rate (exploration rate=5) influences the variability of the generated movements.

#### 3.1.2 PIBB: policy improvement with black box

We implemented the Policy Improvement Black-Box (PI^BB^) algorithm for learning purposes Stulp and Sigaud (2012).

Algorithm 1 PI^BB^: Policy Improvement with Black Box combined with ProMPs.

**Require:** p(θ)∼N(μ,Σ) : Parameter distribution
**Require:** N : Number of samples
**Require:** η : Eliteness factor
**Require:** α : Covariance decay factor
**Require:** $\gamma_{r},\gamma_{g}$ : Reward and guidance decay factors
**Ensure:** Updated parameter distribution $\left(\mu^{\prime },\Sigma^{\prime }\right)$

1: **Sampling:**
2: **for** i=1 to N **do**
3: $\theta_{i}∼N\left(\mu ,\Sigma \right)$

4:  Compute reward $R_{i}=f\left(\theta_{i}\right)$

5: **end for**
6: **Applying decay:**
7: **for** i=1 to N−2 **do**
8:  **if** i∈guidance **then**
9: $R_{i}\leftarrow R_{i}\cdot \gamma_{g}$

10:  **else**
11: $R_{i}\leftarrow R_{i}\cdot \gamma_{r}$

12:  **end if**
13: **end for**
14: **Normalizing rewards:**
15: $R_{\min}\leftarrow \min\left(R\right),R_{\max}\leftarrow \max\left(R\right)$

16: **for** i=1 to N **do**
17: $R_{i}^{\prime }\leftarrow -\eta \left(1-\frac{R_{i}-R_{\min}}{R_{\max}-R_{\min}}\right)$

18:   **if** i∈guidance or i∈correction **then**
19: $R_{i}^{\prime }\leftarrow 1.3\cdot R_{i}^{\prime }$

20:   **end if**
21: **end for**
22: **Computing weights:**
23: $w_{i}=\frac{e^{R_{i}^{\prime }}}{\sum_{j}e^{R_{j}^{\prime }}}$

24: **Updating the distribution:**
25: $\mu^{\prime }\leftarrow \sum_{i}w_{i}\theta_{i}$

26: $\Sigma^{\prime }\leftarrow \alpha \Sigma$

The PI^BB^ approach optimizes a distribution of ProMP weights based on the user rewards. The functionality of the algorithm was implemented as illustrated in pseudocode Algorithm 1.

When a new sample is added, the policy distribution N(μ,Σ) is updated in several steps. First, a set of new samples is generated by drawing parameter vectors $w_{i}$ from the current distribution:

$$\theta_{i}∼N\left(\mu ,\Sigma \right),\;i=1,\ldots ,N$$

For each sample ProMP weight, a mean trajectory is calculated, and the corresponding reward $R_{i}$ is evaluated. Next, reward decay is applied to previous samples. If there are recent guidance samples, rewards of older samples are scaled by the guidance decay factor $\gamma_{g}$ , otherwise, the standard reward decay factor $\gamma_{r}$ is used:

$$R_{i}\leftarrow R_{i}\cdot \gamma_{g}\;\;\text{if }i\in \text{guidance\_samples}$$

$$R_{i}\leftarrow R_{i}\cdot \gamma_{r}\;\text{otherwise}$$

The rewards are then normalized by scaling them between the minimum and maximum values:

$$R_{i}^{\prime }=-\eta \left(1-\frac{R_{i}-R_{\min}}{R_{\max}-R_{\min}}\right)$$

where guidance and correction samples receive an additional weighting factor:

$$R_{i}^{\prime }\leftarrow 1.3\cdot R_{i}^{\prime }\;\;\text{if }i\in \text{guidance\_samples}\text{ or }i\in \text{correction\_samples}$$

The PIBB weight update is computed by transforming the normalized rewards:

$$w_{i}=\frac{e^{R_{i}^{\prime }}}{\sum_{j}e^{R_{j}^{\prime }}}$$

The new mean of the distribution is then updated using these weights:

$$\mu^{\prime }\leftarrow \underset{i}{\sum }\theta_{i}\cdot w_{i}$$

Finally, the covariance matrix decays to reduce exploration over time:

$$\Sigma^{\prime }\leftarrow \alpha \Sigma$$

This process integrates the newly generated samples into the policy distribution while adjusting the influence of past trajectories.

The reward decay factor $\left(\gamma_{r}=0.9\right)$ is applied to reduce the influence of older samples over time. This ensures that more recent trajectories contribute more significantly to the policy update. A distinct guidance decay factor $\left(\gamma_{g}=0.5\right)$ is applied when new guidance samples are introduced. This factor gradually reduces the influence of older samples more significantly. This ensures that the upcoming movements closely align with the guidance movement. To effectively normalize the rewards, an eliteness parameter (η=10) is used, which scales the weight assignment to favor the highest performing samples. This parameter controls the selection pressure, ensuring that top-performing ProMP parameters have a greater impact on shaping the updated distribution. The **covariance** decay factor (α=0.973) progressively reduces the variance of the Gaussian policy distribution over iterations. This mechanism encourages convergence by refining the exploration space while still allowing for incremental adjustments. In addition, samples labeled as corrections or guidance feedback receive a weight adjustment factor of 1.3, increasing their impact relative to standard samples. This weighting ensures that corrections and guidance cues contribute more prominently to the learning process, reinforcing intended behavior modifications. As a result, corrections have a stronger negative impact. This is because undesirable behavior should continue to be avoided throughout the learning process. One can think of it as speaking the first word, an achievement at the time but one that gradually loses significance. In contrast, touching a hot stove remains something to be avoided, no matter how far learning progresses.

In our study, we also developed a graphical user interface (GUI) to showcase the available modalities and to capture user interactions for our analysis. We configured two versions of our GUI: one that contains only the basic modality (group 1), and another that offers all the supplementary modalities to the participants (group 2), see Figure 2.

#### 3.1.3 User interaction and controls

The GUI for each of the experimental conditions is shown in Figure 2. Users could provide feedback on a preference pair by clicking on the left heart button to indicate that the left movement was preferred or on the right heart button for the right movement. Additionally, users had the option to replay the movements by clicking the “Replay” button. For group 2, clicking the “Save” button marked a movement as a fallback, highlighting the button in white. Movements could also be labeled as “Guidance” by clicking the signpost button or as “Correction” by clicking the eraser button. When a movement was actively marked, the corresponding button was highlighted in white. The exploration could be adjusted using a slider that was initially set at a medium level. The slider allowed users to reduce the exploration to the moon icon or increase it to the binocular icon. Similarly, movement speed was controlled through the same mechanism. By clicking the central “Demonstrate” button at the bottom, users entered the demonstration mode, where they could start, stop, and restart the demonstration. If learning progressed in an undesired direction, users could load the fallback by clicking the “Load Save Point” button. This action immediately restored the fallback and generated two new movements based on it. Upon clicking “Submit,” all markings (e.g., Guidance, Correction) and slider settings were recorded for the generation of the next two movements. After submitting, users saw a grayed-out screen displaying the message “Please look at the robot,” during which they could not interact with the interface. The two newly generated movements were then presented. The GUI implemented a basic form of modality conflict handling. Specifically, when a movement was labeled as *Guidance*, any previous *Correction* label was automatically removed, making the two mutually exclusive. Similarly, labeling a movement as *Fallback* also cleared any existing *Correction* label. In cases where a user attempted to load a fallback trajectory without having previously saved one, no action was taken. However, beyond these basic mechanisms, the system did not enforce additional contextual constraints, which means that semantically questionable actions, such as labeling successful movements as *Corrections* were not actively prevented.

### 3.2 Modalities

In this section, we describe the concrete implementation of the different modalities.

#### 3.2.1 Preference

For the preference modality, two different behaviors are presented from which participants could then mark their preferred ones. We used the reward function:

$$\left\{\begin{array}{cc} \left(100,100\right)\; & Both\;preferred\\ \left(100,-100\right)\; & First\;action\;preferred\\ \left(-100,100\right)\; & Second\;action\;preferred\\ \left(-100,-100\right)\; & None\;preferred \end{array}\right.$$

This approach not only enables the articulation of a preference for one option but also accommodates the expression of preference for neither or both options. Our choice for this implementation was driven, among other factors, by the need for the algorithm to perform rapidly and sample-efficiently. To amplify the differentiation in actions, we increased the disparity between the ProMPs drawn from the distribution, addressing the issue that users struggle to express their preferences when actions are too similar. Additionally, we created a significant contrast between positive and negative rewards to make negative ProMPs even less likely. We also opted for samplewise updates to the sampling instead of batchwise, as users in direct interaction expect immediate responses Vollmer and Hemion (2018).

#### 3.2.2 Supplementary modalities

##### 3.2.2.1 Guidance

Was achieved by significantly increasing the influence of the action chosen as guidance in the selection of future actions, while drastically diminishing the impact of all other actions. This is achieved by influencing the decay of rewards by increasing it; this only leaves the last sample marked as guidance. Furthermore, the influence of guidance samples on the mean is increased. Lastly, the guidance samples receive higher rewards of 150 instead of 100.

##### 3.2.2.2 Correction

Unlike *Guidance* samples, *Correction* samples do not introduce additional decay, as negative actions should retain their influence in contrast to previously positive actions. Moreover, as *Guidance*, their impact on the mean is amplified. However, since they receive negative rewards, this amplification occurs in the opposite direction, effectively enhancing their influence negatively. Additionally, *Correction* samples incur a greater penalty, receiving a negative reward of −150 instead of −100 .

##### 3.2.2.3 Demonstrations

Were performed through kinesthetic teaching and utilized as a new average for the new distribution of the PIBB algorithm. Given that users anticipate behavior that closely mirrors the demonstration, past feedback is disregarded; otherwise, movements, particularly in the later stages of the learning trajectory, would deviate significantly from the demonstration due to the influence of previous actions.

##### 3.2.2.4 Exploration

Was facilitated by adjusting the base exploration factor upward or downward on five levels, ensuring, however, that exploration consistently decreases over time.

$$\alpha =\lambda \cdot \epsilon_{0}\cdot 0.96^{r}$$

##### 3.2.2.5 Speed

Was realized as accelerating or decelerating of all movements after the trajectories have been generated, eliminating the need to incorporate an additional dimension into the learning process.

##### 3.2.2.6 Fallback

Was implemented by marking a trajectory, more specifically the ProMP weights of this trajectory, as mean that users can return to as needed. When a fallback is marked, it is also defined as a guidance as well. This triggers all positive effects of a guidance, since a fallback sample is marked as the best so far by the user. The marking as guidance leads to a more positive evaluation, resulting in a score of 150, and influences the further calculation of the distribution as described in the guidance section. If the user returns to the marked action, it has the same effect as providing a demonstration. This means that when loading a fallback, the previous progress is discarded, except for the reduction in exploration, and the learning process continues with the loaded fallback as the new mean of the distribution. The decision for this implementation was based on the users’ expectation of similar behavior to the saved trajectory, rather than the distorted behavior that would arise if past actions were incorporated into the calculation of the next movement. This distortion would also happen, even if the fallback was set as the new mean and the old actions were kept. Thus, all old actions must be discarded. Additionally, it is based on the fact that users want the progress made after the fallback action to be discarded when loading.

### 3.3 Study design

#### 3.3.1 Participants

Participants were recruited on campus through flyers, posters and mailing lists and received a compensation of 10 €. We recruited participants who were considered *lay users*, defined as individuals without prior experience with the specific algorithm or the robot used in the study. This approach reflects our aim including a representative sample of potential end users, such as those who might encounter robots in everyday or care-related environments. All subjects gave their informed consent for inclusion before participating in the study. The participant cohort for the study was randomly assigned to two groups.

#### 3.3.2 Task

**FIGURE 1:**
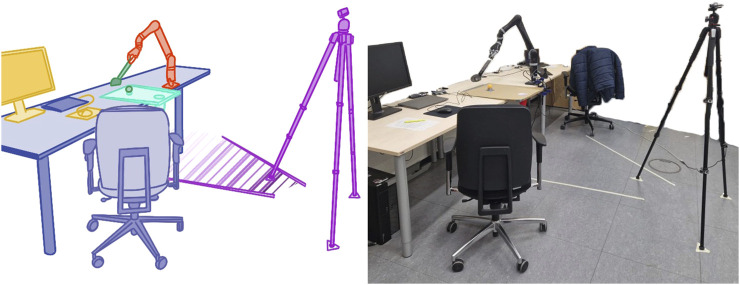
Study setup. Red depicts the Kinova Jaco 2 robot platform, green the task relevant objects, purple the camera for study recording, blue the workspace environment and yellow the user interface.

The task designed for the study was a minigolf challenge without obstacles, chosen for its balanced blend of enjoyment, clarity, and practicality. The setup of the task is depicted in Figure 1. The simplicity of the objective made the goal and proper technique apparent for evaluation. Additionally, the task focuses on the actual execution of the trajectory rather than the target, as seen in tasks like pick-and-place. Therefore, there is no clear or optimal way to do it. At the same time, it offers a wide range of possible execution methods, which can vary significantly between participants. This was already demonstrated in previous studies. An example from the previous studies includes participants who, despite the clear instructions regarding the task’s goal, preferred to execute the task smoothly with a graceful swing rather than focusing on hitting the target. In contrast, others were more focused on fulfilling the task itself and preferred lowering the racket to the playing surface followed by pushing the ball into the hole, although this resulted in much more abrupt trajectories. Furthermore, the straightforward nature of the task allowed for easy reset between attempts. In this process, robot action pairs were evaluated 40 times, even if the task had been completed beforehand. The participants were instructed to succeed (i.e., the robot hits the hole) as often as possible. A trial was considered successful only if the ball was hit directly from a predefined starting position into the hole in a single stroke. The attempt was not counted as successful if the ball touched the boundary. After each action, if the ball was hit, it was reset to the starting position. Both runs were initialized identically using a poor demonstration of the task, which accelerates the algorithm, by indicating the basic nature of the movement (a forward swing) while still preserving the need for learning and human intervention. Moreover, the arm’s reset position was the same for both groups and for every trial. Each task trial proceeded as follows: Participants were shown the GUI with a black overlay and asked to observe the robot. Then, the first movement was presented. After the movement was executed, the robot was reset and the setup was restored. The participant was then informed that the second movement would follow, which was subsequently initiated. After the second movement, the GUI was activated, allowing the participant to provide a rating while the setup was restored again. Once the rating was submitted, this process was repeated.

It was possible to teach the task in very few movements (n<20) . This was achieved through a strategy that involved the use of demonstrations and providing guidance when approaching the ball. Exploration was only reduced after a success. Additionally, reinforcing successful hits with positive rewards, using fallback storage, and strictly penalizing deviations through corrective feedback contributed to efficient learning.

Despite the possibility of completing the task more quickly, we decided to set a fixed number of task repetitions, opting for a notably higher count of 40 trials. The rationale behind this fixed number is to ensure comparability between participants and to gather a similar amount of input from each. The choice of 40 trials was based on previous experiments, which showed that participants were able to achieve at least one success within this timeframe, even with a more restrictive approach. This success left participants with a positive feeling about the study and ensured that all participants completed all 40 trials without opting to quit.

#### 3.3.3 Procedure

**FIGURE 2:**
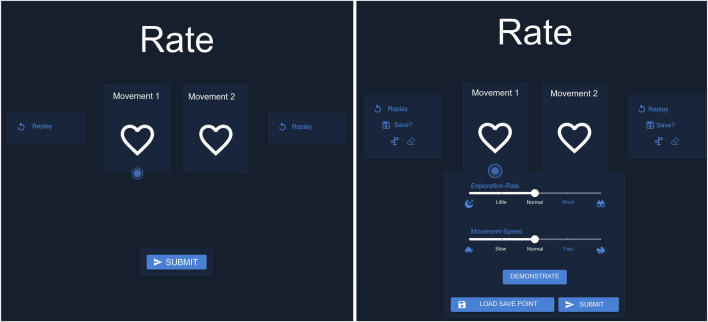
This Figure showcases the translated version of the interface for group 1, with only the preference selection the baseline version and group 2, equipped with all combined modalities the multimodal version (translated from German).

The study began with a welcome and briefing on the task of teaching a robotic arm to play minigolf. The protocol detailed the use of a user interface for rating the robot’s task executions. The GUI for group 1 was designed for a simple interaction; see Figure 2 left. It displays two options, movement 1 and movement 2, each with a replay button and a heart symbol for expressing preference. At the bottom, the participants had a submit button to confirm the choices. The second GUI for group 2 was configured to evaluate robotic movements with all modalities, see Figure 2 right. In addition to the features available to group 1, it also has the option to save movements as fallback. Furthermore, central sliders allow users to adjust exploration rates and movement speed. The central button on the bottom facilitates the demonstration of movements, and on the right there is the option to load a fallback movement.

In the description of the individual interfaces, the conditions of each element were explained to the users, along with their effects, such as how Guidance marks a trajectory as a reference. However, the internal implementation of these effects within the algorithm was not described. The different modalities were clearly differentiated in their function and the corresponding UI elements were highlighted. For example, it was explained that saving a fallback allows users to return to the saved state and that Guidance, as described, marks the movement as a reference. However, it was not mentioned that Fallback internally utilizes the functionality of Guidance. Since the study investigated how lay users interact with these modalities and since users were not aware of how such mechanisms work internally, an introduction to the algorithmic implementation was omitted. After the introduction to the corresponding GUI, the experimenter outlined the recording of choices and timing for analysis purposes, concluding with a post-interaction questionnaire described below and compensation details. After receiving the above information, the participants were asked for their consent to participate in the study, as well as for the recording and use of their data. They were explicitly informed of their right to withdraw from the study at any time or request the deletion of their data.

#### 3.3.4 Data acquisition and analysis

According to the hypotheses and aims of the study, the data collection focused on two main objectives: evaluating learning progress and assessing user satisfaction and preference.

The learning process was analyzed through video recordings of the playing field. Our primary metric for assessing learning progress was the completion of the task, specifically measuring how often the task was completed successfully and the timing of the first success. Success was defined as the ball being hit by the robot with the club and going directly into the hole without touching the edges. In addition to comparing the total number of successes, we also looked at individual movements (1–80), noting the percentage of participants in each group that scored a successful hit on the try. In this study, we focused on task success and first hit as primary evaluation metrics, since the objective was to understand how different feedback modalities influence the learning process. Importantly, task success and first hit offer a consistent basis for comparison across highly diverse teaching strategies and the resulting trajectories. Given the variability in user behavior and the flexibility of interaction design, these outcome-oriented metrics allow us to meaningfully relate modality usage to learning effectiveness, regardless of how the behavior was taught. These hit success metrics were measured between groups, with the preference rating serving as the baseline modality for both groups. Group 1 only had access to the base modality, while group 2 also had access to supplementary modalities 2. This setup allows for assessing the impact of extra modalities on learning in a between-subjects design.

In terms of user satisfaction, overall satisfaction with the system was measured for both groups on a 5-point Likert scale. The metric was also compared between groups using the same question, generally referring to the system and not to specific modalities, making this a between-subjects design as well. We calculated significances across groups to analyze the data.

In addition to the between-subjects design, we also had research questions that focused solely on group 2, which had access to the supplementary modalities, such as their preferences and perception of these modalities. Therefore, the following research questions were addressed using a within-subjects design.

Group 2 participants also responded to the System Usability Scale (SUS) questionnaire Brooke (1995) for each supplementary modality. From the calculated SUS scores, the average was determined for each supplementary modality and compared pairwise. The preferences for the modalities were measured not only through SUS evaluations but also directly through the ranking of the supplementary modalities. We also collected the reasons behind these rankings. For each option in the ranking, we calculated the percentage frequency of its placement in each position. The qualitative statements of the participants were analyzed by categorizing them into thematic groups and then evaluating these categories according to their frequency. Lastly, we analyze the relationship between learning success and modality usage. To assess differences between the two experimental groups, we applied Kruskal–Wallis tests for all between-subject comparisons, given the nonparametric nature of the data. For within-subject analyses, such as the evaluation of user preferences across multiple modalities, we used Friedman tests. When significant effects were detected, we conducted Conover’s all-pairs post-hoc tests with Holm correction for the between-subject and Wilcoxon signed-rank tests for the within-subject analysis. All questionnaire items for group 1 and group 2 can be found in the Supplementary Appendix.

### 3.4 Ethics declarations

The research design for this study was reviewed and approved by the local ethics committee of Paderborn University in the scope of TRR 318 Constructing Explainability. Participants gave their informed consent before the experiment.

### 3.5 LLM usage

ChatGPT or similar tools were only used to improve the text syntactic and grammatical. All suggestions made by the AI tools were thoroughly checked by all authors.

## 4 Results

We now present the results of our study, structured around our hypotheses. Our study involved 33 German-speaking participants divided into two groups, with group 1 “baseline” using a basic interface to express preferences between two movements and group 2 “multimodal” having access to the described additional supplementary modalities. group 1, consisted of 15 individuals: 6 male, 8 female, and 1 identifying as diverse. group 2 comprised 18 participants: 9 male and 9 female. Across groups, there were individuals (12 in group 1, 9 in group 2) with a higher education entrance qualification and 13 (3 in group 1, 9 in group 2) with a higher education degree, totaling 33 participants. All 33 participants were German-speaking, aligned with the German-only interface of the system used. A detailed description of the implementation, modalities, tasks, procedure, and data analysis is provided in Section 3. We begin with the evaluation of learning success H1.

**FIGURE 3:**
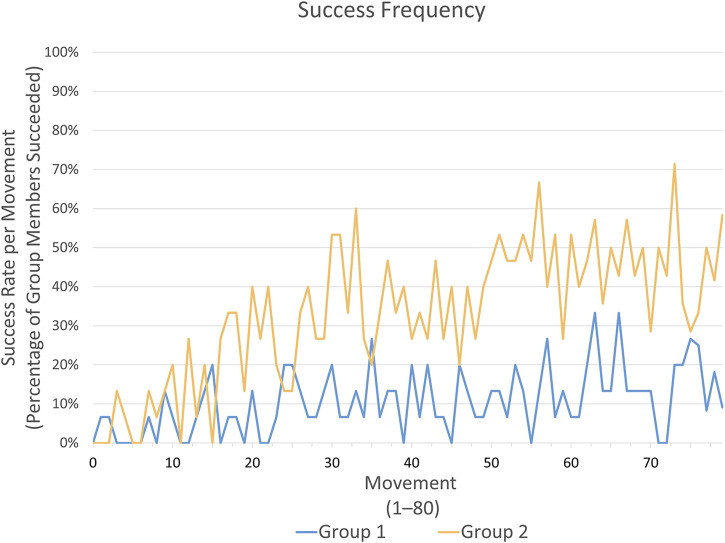
This graph illustrates the percentage of success per movement (80 movements resulting from 40 trials with 2 movements each) across all participants within each group.

Comnparison of the total number of successful hits between group 1 “baseline” and group 2 “multimodal” showed a significant difference in the performance of the two groups, with group 2 “multimodal” successfully hitting more frequent. A Wilcoxon rank-sum test found a significant difference between group 1 (Median =5.0 , Mean =8.33 , SD =9.80 ) and group 2 (Median =26.5 , Mean =25.56 , SD =15.62 ) (W =38 , p =0.0013 ). Cliff’s Delta (Δ=−0.683,95% CI: [−0.89,−0.22]) indicates a large effect, with group 2 consistently showing higher values. The results are visualized in Figure 3, which presents the percentage of participants who were successful in each movement, divided into the two groups.

Participants in group 2 “multimodal” achieved their first hit on average after 17.56 ((Median = 16, SD = 13.65)) attempts, while participants in group 1 “baseline” required 34.60 (Median = 26, SD = 27.93) attempts to reach their first successful hit. A Wilcoxon rank-sum test found a trend toward a difference between group 1 and group 2 ( W=164.5 , p=0.0814 ). Cliff’s Delta (Δ=0.371, 95% CI: [−0.06,0.69]) suggests a moderate effect, with group 1 tending to show higher values.

Next, we will present the participants’ usage preferences for the supplementary modalities to answer the second hypothesis H2. The general satisfaction level of group 1 “baseline” was consistently moderate, with M=3.31 ((Median =3.5,SD=1.14 ). The mean score for overall satisfaction in group 2 ″multimodal” was notably higher, at M=4.17 (Median =4.0 , SD=0.857 ). A Wilcoxon rank-sum test found a significant difference between group 1 and group 2 (W=80.5,p=0.0223) . Cliff’s Delta (Δ=−0.441, 95% CI: [−0.71,−0.06]) indicates a moderate effect, with group 2 tending to show higher values. Due to the demonstration modality being sparsely (n = 2) used, we excluded it from the evaluation of user preference and utilization, as the results would not be representative. To assess the usability of the user interface and ensure that its design does not hinder users, as well as to evaluate learnability, participants were asked to complete the System Usability Scale (SUS) questionnaire. On a scale ranging from 0 to 100, SUS scores interpret usability and learnability. With mean SUS scores of M=79.17 (Median =79.16667,SD=8.83 ) for *Guidance*, M=79.67 (Median =80.00,SD=14.81 ) for *Speed*, M=74.22 (Median =75.00,SD=15.80 ) for *Correction*, M=72.67 (Median =72.66667,SD=12.12 ) for *Fallback*, and M=68.82 (Median =68.82353,SD=16.94 ) for *Exploration*, the system is generally perceived as ’good’ in usability. A Friedman test found a significant difference in SUS scores across modalities $\left(\chi^{2}\left(4\right)=17.78,\;p=0.0014\right)$ . Post-hoc Conover’s all-pairs test (Holm correction) revealed significant differences, particularly between Exploration and Guidance ( p=0.0080 , r=0.513 , large) and Exploration and Speed ( p=0.0023 , r=0.727 , large). The medians of the groups ranged from 68.82 (Exploration) to 80.00 (Speed), with Speed and Guidance scoring the highest. No significant differences were found in the other pairwise comparisons presented in Table 1.

**TABLE 1:** p-Values of pairwise asymptotic Friedman Test with $\chi^{2}=17.777$ , df=4 , p−value=0.00136 .

	Correction	Exploration	Fallback	Guidance
Exploration	0.1288	-	-	-
Fallback	0.6544	0.8035	-	-
Guidance	0.8035	0.0080	0.0821	-
Speed	0.6544	0.0023	0.0299	0.8035

**FIGURE 4:**
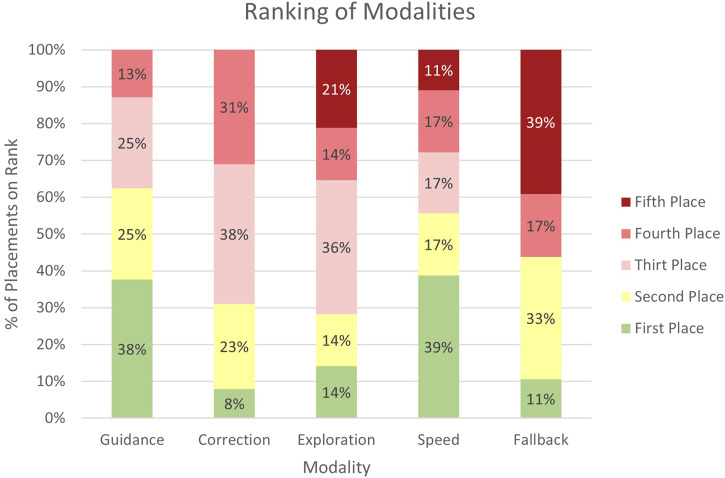
This diagram showcases the ranking distribution of the supplementary modalities. It illustrates the percentage placement of each supplementary modality across the ranking positions by group 2 “multimodal” participants.

Furthermore, participants were asked to rank the supplementary modalities according to their overall satisfaction, with the most popular ones placed at the top and decreasing in popularity from there. We have illustrated the results of the ranking of supplementary modalities in Figure 4, showing the proportion of rankings for each modality each time it was ranked. The results indicate a preference for the *Guidance* modality, which was placed mainly in the first and second rank. *Speed* is favored as well, with a similar portion of its first rank, although it also shows a spread across third to even fifth ranks in contrast to *Guidance*. The *Correction* modality is ranked with moderate preference, apparent from its distribution mostly between the second to fourth ranks, indicating a balanced reception. Similarly, *Fallback* shows a slight lean towards the first rank but also towards the fifth rank. *Exploration* has a majority of its rankings in the lower fourth and fifth ranks. Overall, *Guidance* and *Speed* were the most liked, while *Exploration* is far behind in the ranking.

**FIGURE 5:**
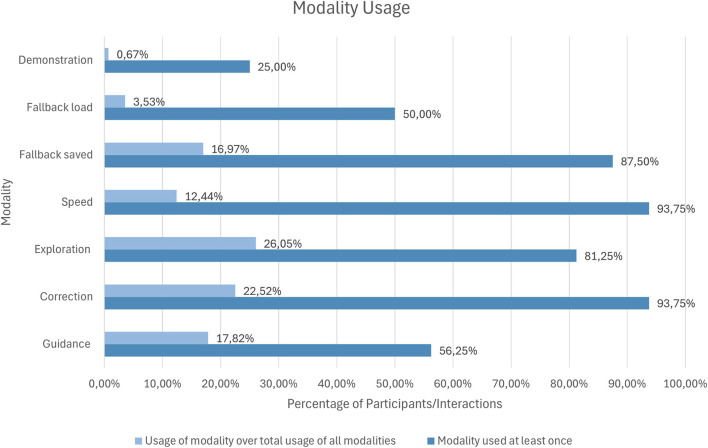
This Figure shows two perspectives on modality usage. It displays how often each feedback modality was used across all interactions of all participants and how many participants used each modality at least once. This allows for comparing overall usage frequency with how widely each modality was adopted, highlighting whether some modalities were broadly preferred or mainly used by a few participants.

We evaluated two key metrics for usage frequencies: a) The total usage means per participant where a modality, when utilized, is counted once; b) The relative count which aggregates across all participants, measuring how frequently each modality was used overall. The results are displayed in Figure 5.

The *Demonstration* modality saw minimal use, with just 0.67% of all interactions and only 25% of participants using it. However, most of the participants initiated this interaction but stopped before performing a kinesthetic teaching demonstration. Only n=2 participants completed and submitted a demonstration. The *Fallback* modality consists of two parts; saving of the fallback point and the load of such a point. In contrast, *Fallback load* was used by a higher percentage of participants (50%) , despite accounting for only 3.53% of all interactions. On the other hand, *Fallback saved* was not only used by a majority (87.5%) of participants but also represents a higher portion of interactions at 16.97% . *Speed* stood out with the highest engagement, utilized by 93.75% of the participants and making up 12.44% of all interactions. *Exploration* was also popular, with 81.25% of the participants engaging with it and 26.05% share of total modality use. Similarly, *Correction* was used by the same percentage of participants as *Speed* (93.75%) , but with a higher percentage of interactions with 22.52% participants who used it. Meanwhile, *Guidance* was used by 56.25% of participants and in 17.82% of all interactions. Overall, the *Speed* and *Correction* modalities lead in participant usage, with *Correction* also notable for interaction frequency alongside *Exploration*, whereas *Demonstration* lags behind with the lowest interaction rate and participant engagement.

**FIGURE 6:**
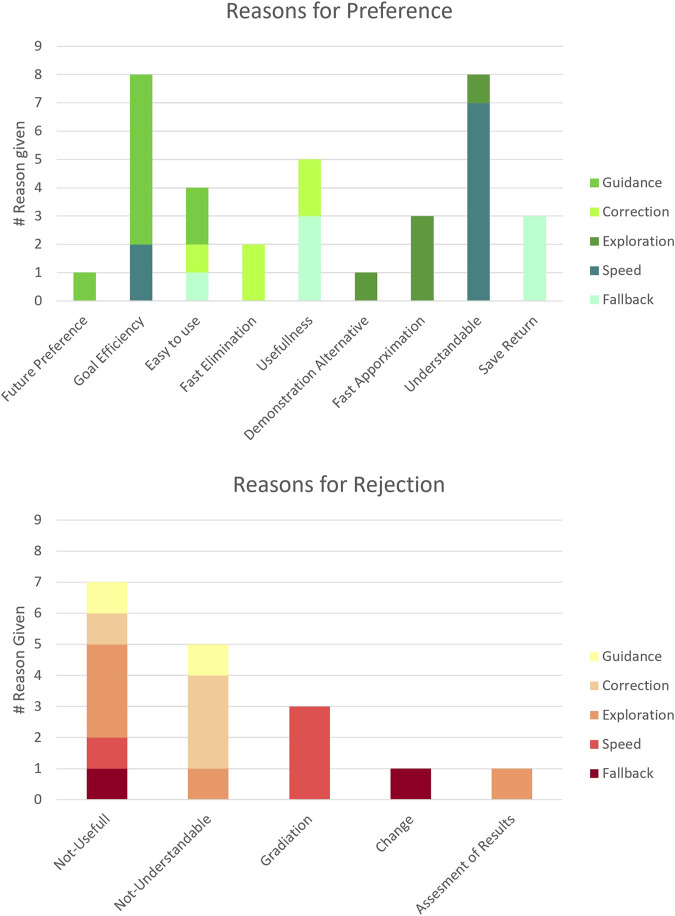
Participants’ reasons for preferring or rejecting supplementary feedback modalities, categorized by modality type and rationale. Positive reasons are shown in green, negative reasons in red. The height of each bar reflects the number of participants who mentioned a given reason, providing a quick overview of participants’ perceptions.

To answer the third hypothesis H3 regarding the perceived value of the modalities, we also captured the participants’ explanations for the rankings analyzed above. These are depicted in Figure 6, categorized into positive justifications for higher placements and critical reasons for lower rankings.

Regarding the perceived use for a positive influence on learning, we considered the reasons “Goal Efficiency”, “Fast Elimination”, “Usefulness” and “Fast Approximation” since these rationals are most closely related to the research question. Guidance was valued for its “Goal Efficiency” and with the most goal-related reasons given. Correction was appreciated for its “Fast Elimination” and its “Usefulness”. *Fallback* was also rated for its “Usefulness”. Exploration was appreciated for its “Fast Approximation”. Regarding the perceived negative influence on learning, the only reason directly related to learning success was “Not-Useful”, which was most frequently noted for the low rankings of the Exploration modality. For all other rankings that are not related to learning, we refer the reader to the Figure 6.

The last hypothesis H4 is concerned with whether some modalities have objectively more influence on robot learning. Spearman’s rank correlation was used to analyze the correlations of hits per try and modalities usage per try. A correlation between *Exploration* and hit success revealed a statistically significant, albeit weak, negative correlation with (ρ=−0.21,p=0.025) . Thus, with a decrease of the exploration rate, the likelihood of hitting the target marginally increases. The analysis of *Speed* revealed a modest positive correlation (ρ=0.2389221,p=0.03898) . This suggests that a higher *Speed* value correlates with a slightly better chance of hitting the target. In contrast, the correlation with the load (ρ=−0.01) and the save (ρ=0.007) of a fallback was not only very weak but also lacked statistical significance with (p=0.7) and (p=0.8) . Guidance also showed a weak negative correlation with hit success, with (ρ=−0.03) , which was also not statistically significant (p=0.3) , suggesting that *Guidance* does not significantly influence hit success. Between *Correction* and hit success, a statistically significant negative correlation ( ρ=−0.07 , p=0.01 ) was found. However, the correlation is below 0.1, and therefore cannot even be considered weak. Overall, the analyses show that while an increase in *Speed* and a decrease in *Exploration* positively influence hit success, *Fallback*, *Guidance*, and *Correction* show no significant or notable correlations with hit success. In the following section, we discuss these results answering our research question.

## 5 Discussion

In the discussion, we will review and address each hypothesis.

**FIGURE 7:**
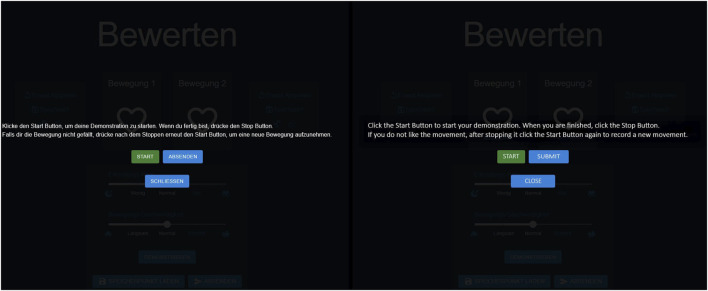
Original (German) and translated (English) versions of the demonstration interface shown side by side. It overlays the regular interface when the *Demonstration* button is clicked.

H1: Enhanced Learning through Multiple Modalities. Our results affirm that simultaneously providing multiple feedback modalities significantly enhances the learning process, as evidenced by the higher performance of group 2 “multimodal” with respect to total success. This group was significantly more successful with respect to the number of hits and showed a clear trend in succeeding faster for the first time. Both confirm that multiple modalities not only enhance initial learning success, but also increase further learning progress. This might be connected to the fact that users were able to actually apply their scaffolding teaching strategies.
H2: User Preferences and Modality Utilization. Generally, the users preferred the system with the combined modalities. This is evident by the higher overall satisfaction observed in group 2 “multimodal”. Combined with the positive SUS score results for the modalities, this suggests that the design of the user interface is not hindering users. In addition, the results of the SUS questionnaires indicate that the learnability of all modalities was rated between moderate and good. Although the SUS questionnaire is not a classical measure for assessing interface complexity, it nevertheless provides useful insight into users’ perceived complexity particularly through items 2 (”*I found the system unnecessarily complex.*”), 3 (”*I thought the system was easy to use.*”) and 4 (”*I think that I would need the support of a technical person to be able to use this system.*”). These items indirectly capture how intuitive and easy to learn the interface and its modal interactions appear to users. In this study, the reported means ( M2=2 , M3=4 , M4=2 ) and medians ( Mdn2=1 , Mdn3=5 , Mdn4=1 ) on these items suggest that users generally did not perceive the modalities or their interface representations as too complex or difficult to use. However, this only captures the complexity of each individual modality; in the future, measures such as NASA-TLX should be included to assess the cognitive load of combining multiple modalities. Furthermore, our results show that users do not engage with all modalities equally. The modalities favored by the participants were reflected in their frequency of use, ranking, reasons provided for their choices, and SUS scores for each supplementary modality. Notably, *Guidance* and *Speed* stand out for their high use and positive reception, attributed to their perceived impact on the learning process and intuitiveness. Both also stand out in the pairwise comparison regarding the SUS score compared to *Exploration*. In contrast, *Exploration* and *Demonstration* were less favored. The former was criticized for its ambiguous effects. For the latter, the lower preference is evident in the lack of engagement with the *Demonstration* modality. Despite the explicit introduction of the *Demonstration* modality in the introduction of the study, the use of hover-over tool tips, the prominent placement of the demonstration button (cf. Figure 2), and a simple demo interface that resembles a recorder (see Figure 7) including an additional explanation of the process whenever participants started the demonstration, the participants’ engagement with the modality remained limited.

The sparse interaction might be caused by the perceived effort or lack of necessity also found in the literature regarding the usage of demonstration in HIL-RL Casper et al. (2023); Brown et al. (2019). It might also be the case that participants could not assess the value of the demonstration. The exact function of the algorithm was not explained in the context of the study and the necessary expertise was not necessarily present. In addition, the participants might have found the robot intimidating. Combined with the increased physical effort required and the perceived comparable value of other modalities, their preference for familiar functions like rating with hearts or icons for preferences, as well as modalities with predictable outcome as the *Speed* modality, may have outweighed the demonstration’s benefits. Moreover, the presented setup of multiple simultaneously available modalities has rarely been studied, and understanding the behavior and preferences of participants in this context is highly relevant. Thus, the neglect of well-established modalities should be explored further in future research. An additional noteworthy observation concerns the use of the *Fallback* modality. Fallback saving may have been used more frequently than loading because participants might have aimed to preserve their progress and may have seen little need to return to a previously saved state. Saving a fallback point might have been associated with a predictable and positive outcome, it marked a successful movement, and entailed no risk to the current learning state. In contrast, loading a fallback point could potentially disrupt the ongoing learning trajectory, introducing uncertainty, and requiring stronger commitment from the user. Participants may have hesitated to risk overwriting progress by returning to a prior state.

H3: Different Perception of Modalities. Our findings support the hypothesis that users perceive certain modalities as more beneficial than others in improving the learning process when choosing. *Guidance*, with its high engagement and favorable ranking, was considered particularly effective, highlighting its role in achieving learning goals efficiently. Although *Correction* and *Fallback* were recognized for their utility, their engagement levels and mixed rankings suggest a nuanced perception of their effectiveness in learning enhancement. Both *Correction* and *Fallback* may have ended up in the middle range of preference because they are primarily used in response to errors or setbacks, that is, in situations where the learning or teaching process is not going as intended. However, the overall learning process may have progressed smoothly for many participants, reducing the perceived need to engage with these corrective strategies. *Guidance*, on the other hand, may have gained popularity precisely because it represents a form of positive feedback, reinforcing successful actions, and supporting learners proactively rather than reactively. *Exploration* was frequently used and valued for fast approximation; however, it was also often labeled as “Not-Useful” and received the lowest ratings, making it one of the underutilized modalities. The perception of Exploration may have suffered not only from the lack of immediate transparency but also from the prerequisite of a certain level of underlying understanding required to use it effectively. *Demonstrations* were used very rarely, indicating a low preference among participants. The low engagement with the *Demonstration* modality may be explained by factors outlined in H2, such as the perceived physical effort involved, a lack of understanding of its added value and the availability of effective alternatives. Hence, when given a choice, these modalities are likely to be dismissed in favor of others.
H4: Enhanced Learning through Individual Modalities. Our results have shown that *Exploration* and *Speed* have a positive impact on hit success. Here, *Exploration* needs to be reduced upon successful hits to maintain performance, and *Speed* is required to be increased to ensure the ball’s reach. Although *Exploration* was seen as the least useful and preferred modality, the results indicate that when used effectively (decreasing on success and increasing in the beginning), it positively influences learning success. Movement attributes such as *Speed* are perceived positively, frequently interacted with, understandable, and directly influence learning success. Thus, they appear to represent a natural teaching method for participants in this task context, eventhough they are underestimated in the field Tien et al. (2024). In contrast to modalities such as *Speed*, which were frequently used and positively correlated with learning success, and others like *Exploration* or *Demonstration*, which showed or are known for a potential positive effect but were underutilized, there was also *Guidance* that was highly favored by participants but did not show statistically measurable benefit for learning outcomes. The high subjective rating of *Guidance* may stem from its association with already successful movements, leading to a positive memory bias, as well as from the strong sense of control it offers users during interaction. However, its effects are delayed and may not manifest within the same trial, making them difficult to capture with a binary success metric. Additionally, inconsistent usage across participants and interaction with other modalities may have further diluted its measurable impact. A similar pattern can be observed with *Correction*. This may be partially due to the lack of contextual constraints in the system, which allowed users to provide semantically inconsistent feedback, for example, labeling actions as corrections even when they brought the robot closer to the intended goal, or marking actions as guidance based on higher-level intent rather than concrete trajectory advantages, which may not have been beneficial for the learning process. In this study, we intentionally did not restrict users from such feedback behavior to explore whether users would engage in counterproductive labeling in a free-form multi-modal environment. The findings of the positive perception and low effect suggest that future systems should consider offering greater transparency or explanation regarding how each modality influences the learning process. Providing lightweight scaffolding or contextual cues could help users better understand the consequences of their feedback without compromising their autonomy or limiting natural teaching strategies.

The diversity of strategies observed among participants resulted in highly varied patterns of modelity usage, making it difficult to identify consistent correlations between specific combinations of feedback modalities and learning success. However, this lack of clear patterns does not necessarily imply an absence of underlying structure. Prior work by Vollmer et al. Vollmer and Hemion (2018) has shown that even when users are restricted to a single modality, they employ a wide range of teaching strategies, highlighting the inherently individualized nature of human teaching behavior. Furthermore, studies such as Bıyık et al. (2022) and Jeon et al. (2020) suggest that the effectiveness of human feedback is influenced by their timing, frequency, and contextual fit. These findings underscore the need for adaptive systems that go beyond fixed modalities. Our findings, in combination with these prior work, suggest that the mixed and inconsistent use of different modalities may reflect the fact that offering a wide range of feedback options accommodates diverse teaching strategies. From this perspective, the absence of clear usage patterns is not necessarily problematic; in fact, it may indicate that users are flexibly selecting modalities according to their individual preferences. In contrast, the presence of strong patterns could point to limitations in the accessibility or usability of certain modalities. However, it is also possible that clearer correlations between modality use and learning success would have emerged if modalities had been offered more flexible and adaptively, in line with user needs. This points to an important direction for future work: exploring dynamically personalized modality provision to better support different instructional approaches.

### 5.1 Limitations

Despite the promising results of our study, several limitations must be acknowledged that may affect the generalizability and interpretation of our findings.

First, although we evaluated the usability of each modality using the System Usability Scale (SUS), this instrument was not originally designed to assess cognitive load. As such, it provides only an indirect approximation of the cognitive load induced by the complexitiy of the interface. Future work should incorporate dedicated measures such as NASA-TLX to gain a more precise understanding of the cognitive load caused by using multiple modalities simultaneously.

Second, the *Demonstration* modality, despite being introduced and available, was used very rarely. While this finding aligns with the prior literature as described above, it limits the interpretability of our results for this modality. Low usage may result from perceived physical effort, intimidation by the robot, or a lack of understanding of its benefit. For HIL-RL, this highlights the need for targeted explanation and transparency mechanisms that clarify the added value of each modality. Those scaffoldings should be investigated particularly for modalities with high investments and implicit effects like *Demonstration* and *Exploration* in order to offset effort and hesitation, and to support more informed and balanced modality usage.

Third, our study was conducted in a simple learning scenario, since this setup allowed a controlled comparison of user strategies and modality preferences. This limits the generalizability of our findings to more complex task domains. It remains unclear whether the observed preferences and usage patterns would hold under these more complex conditions. Moreover, user preferences may shift. This limitation also extends to the learning algorithm itself, if it would perform effectively in more complex task domains. Additionally, it is an open question whether the learned policy would generalize well to different contexts beyond the original training setting. Future work should therefore investigate how the combined use of feedback modalities in complex environments and whether additional scaffolding, constraint mechanisms, or adaptive interfaces are needed to maintain effectiveness and usability.

Fourth, the diversity of teaching strategies observed among the participants made it difficult to identify consistent correlations between the use of individual modalities and the learning outcomes. This highlights the need for adaptive and personalized modality presentation, which can dynamically respond to user behavior to support more consistent learning progress.

Taken together, these limitations do not undermine the core findings but rather highlight important directions for refining experimental design, user interfaces, and system adaptivity in future research on multi-modal human-in-the-loop robot learning.

## 6 Conclusion

Overall, we conduct a study to explore the combined use of multiple feedback modalities in HIL-RL to improve robot learning and user satisfaction. The novelty lies in examining how lay users interact with various simultaneously offered feedback modalities identifying their benefit and usage preferences. In summary, we found that offering a variety of feedback modalities does not hinder users, but rather enhances the learning process of the system. Additionally, our results show that even in a relatively simple task environment, providing multiple feedback modalities can positively influence the learning process. While the observed improvements are based on a specific learning framework and task setting, the underlying approaches (PI^BB^, ProMPs) are conceptually transferable. This study suggests that similar patterns in the use of modalities and teaching strategies could likewise emerge in more complex or less structured tasks, indicating a potential for broader applicability. Although this remains to be empirically validated, it highlights the relevance of considering simultaneous and diverse multimodal interaction early in the development of interactive learning systems, especially in domains where user guidance and adaptability play a critical role. Furthermore, we observed that users have preferences for certain modalities that do not always align with the measurable contributions of each modality to the learning process. We thus advocate for a broader variation and integration of multiple simultaneously provided modalities within the domain of interactive robot learning. In our selection of modalities, our results show that, for example, clarifying *Exploration* could result in even more significant learning improvements. Thus, our second call for further research is to focus particularly on transparency and explaining modalities. This especially seems to hold for modalities such as *Exploration* without a directly observable effect, as,for example, for *Speed*. This allows users to utilize the modality that best meets their individual teaching and scaffolding strategies and preferences. Regarding the question of modality stacking, related frameworks such as DemPref by Bıyık et al. (2022) suggest that diminishing returns of modality usage are not necessarily caused by the number of modalities themselves, but rather by when and how extensively they are employed. Their findings show that demonstrations, for instance, offer substantial value early on but become less informative with increased usage, whereas preferences are more fine-grained and useful in later phases. Jeon et al. (2020) also do not define diminishing returns in terms of the number of modalities used. Instead, they suggest that the frequency and context of modality use are critical factors influencing the informativeness of human feedback. Consequently, rather than emphasizing a fixed set of modalities, the frameworks highlight the importance of flexibility and adaptivity, advocating for systems that dynamically adjust the frequency of the feedback modelities. This indicates that beyond parallel application, systems should offer greater flexibility and adaptivity by dynamically selecting modalities based on context and user needs. Moreover, future frameworks should provide explicit guidance and constraints regarding the frequency of modality use, with the goal of supporting users more effectively and preventing cognitive overload.

## Data Availability

The raw data supporting the conclusions of this article will be made available by the authors, without undue reservation.

## Supplementary material

SUPPLEMENTARY FILE 1 Questionnaire group 1 (translated from German).

SUPPLEMENTARY FILE 2 Questionnaire group 2 (translated from German without demonstration SUS questionnaire).
