# Supplementary material for: The power of combined modalities in interactive robot learning
Source: Front Robot AI. 2025 Jul 17;12:1598968. doi: 10.3389/frobt.2025.1598968 (PMC12312635; doi:10.3389/frobt.2025.1598968)
Supplement: Supplementary file 1 [file Supplementaryfile1.pdf]

soSci
oFb - der onlineFragebogen

Seite 01

1. Gender inquiry

What is your gender?

- ◯ Male
- ◯ Female
- ◯ I define myself differently

2. Educational qualification

What is your highest level of education?
Please select the highest level of education you have attained.

[Please select] ⌄

Seite 02

3. Satisfaction with the system

Please rate your satisfaction with the overall system.

	Very dissatisfied				Very satisfied
Satisfaction	◯	◯	◯	◯	◯
