# Supplementary material for: The power of combined modalities in interactive robot learning
Source: Front Robot AI. 2025 Jul 17;12:1598968. doi: 10.3389/frobt.2025.1598968 (PMC12312635; doi:10.3389/frobt.2025.1598968)
Supplement: Supplementary file 2 [file Supplementaryfile2.pdf]

soSci
oFb - der onlineFragebogen

Seite 01

1. Gender inquiry

What is your gender?

- ○ Male
- ○ Female
- ○ I define myself differently

2. Educational qualification

What is your highest level of education?
Please select the highest level of education you have attained.

[Please select] ⌄

Seite 02

3. Satisfaction with the system

Please rate your satisfaction with the overall system.

	Very dissatisfied				Very satisfied
Satisfaction	○	○	○	○	○

4. Satisfaction with the undo function

	Strong rejection				Strong agreement
I think that I would like to use the undo function frequently.	○	○	○	○	○
I find the undo function unnecessarily complicated.	○	○	○	○	○
I find the undo function easy to use.	○	○	○	○	○
I believe I would need the support of a technical person to use the undo function.	○	○	○	○	○
I think the undo function is well integrated.	○	○	○	○	○
I find that the undo function did not behave as expected.	○	○	○	○	○
I can imagine that most people would learn to use the undo function very quickly.	○	○	○	○	○
I find the undo function very cumbersome to use.	○	○	○	○	○
I feel very confident using the undo function.	○	○	○	○	○
I had to learn a lot before I could start using the undo function.	○	○	○	○	○

5. Satisfaction with the guidance function

	Strong rejection				Strong agreement
I think that I would like to use the guidance function frequently.	○	○	○	○	○
I find the guidance function unnecessarily complicated.	○	○	○	○	○
I find the guidance function easy to use.	○	○	○	○	○
I believe I would need technical support to use the guidance function.	○	○	○	○	○
I think the guidance function is well integrated.	○	○	○	○	○
I find that the guidance function did not behave as expected.	○	○	○	○	○
I can imagine that most people would learn to use the guidance function very quickly.	○	○	○	○	○
I find the guidance function very cumbersome to use.	○	○	○	○	○
I feel very confident using the guidance function.	○	○	○	○	○
I had to learn a lot before I could start using the guidance function.	○	○	○	○	○

6. Satisfaction with the correction function?

	Strong rejection				Strong agreement
I think that I would like to use the correction function frequently.	○	○	○	○	○
I find the correction function unnecessarily complicated.	○	○	○	○	○
I find the correction function easy to use.	○	○	○	○	○
I believe I would need technical support to use the correction function.	○	○	○	○	○
I think the correction function is well integrated.	○	○	○	○	○
I find that the correction function did not behave as expected.	○	○	○	○	○
I could imagine that most people would learn to use the correction function very quickly.	○	○	○	○	○
I find the correction function very cumbersome to use.	○	○	○	○	○
I felt very confident using the correction function.	○	○	○	○	○
I had to learn a lot before I could start using the correction function.	○	○	○	○	○

7. Satisfaction with the exploration function

	Strong rejection				Strong agreement
I think that I would like to use the exploration function frequently.	○	○	○	○	○
I find the exploration function unnecessarily complicated.	○	○	○	○	○
I find the exploration function easy to use.	○	○	○	○	○
I believe I would need technical support to use the exploration function.	○	○	○	○	○
I think the exploration function is well integrated.	○	○	○	○	○
I find that the exploration function was too inconsistent.	○	○	○	○	○
I could imagine that most people would learn to use the exploration function very quickly.	○	○	○	○	○
I find the exploration function very cumbersome to use.	○	○	○	○	○
I feel very confident using the exploration function.	○	○	○	○	○
I had to learn a lot before I could start using the exploration function.	○	○	○	○	○

8. Satisfaction with the function for adjusting movement speed

	Strong rejection				Strong agreement
I think that I would like to use the function for adjusting movement speed frequently.	○	○	○	○	○
I find the function for adjusting movement speed unnecessarily complicated.	○	○	○	○	○
I find the function for adjusting movement properties easy to use.	○	○	○	○	○
I believe I would need the support of a technical person to use the function for adjusting movement speed.	○	○	○	○	○
I think the function for adjusting movement speed is well integrated.	○	○	○	○	○
I find that the function for adjusting movement properties did not behave as expected.	○	○	○	○	○
I could imagine that most people would learn to use the function for adjusting movement speed very quickly.	○	○	○	○	○
I find the function for adjusting movement properties very cumbersome to use.	○	○	○	○	○
I feel very confident using the function for adjusting movement properties.	○	○	○	○	○
I had to learn a lot before I could start using the function for adjusting movement speed.	○	○	○	○	○

10. Please rank the input options listed below according to your satisfaction with them. If you have not used a particular input option, do not include it in your ranking.

Input option	Rank
Guidance function	1
Correction function	2
Undo function	3
Adjustment of movement speed	4
Exploration function	5
Demonstration function	6

11. Justification for arrangement

Please justify your arrangement of input options below.
